# Fine Mapping and Gene Analysis of *restorer-of-fertility* Gene *CaRf_HZ_* in Pepper (*Capsicum annuum* L.)

**DOI:** 10.3390/ijms23147633

**Published:** 2022-07-11

**Authors:** Zhixing Nie, Yunpeng Song, Hong Wang, Jianying Chen, Qingliang Niu, Weimin Zhu

**Affiliations:** 1 Shanghai Key Laboratory of Protected Horticultural Technology, Horticulture Research Institute, Shanghai Academy of Agricultural Sciences, Shanghai 201403, China; niezhixing@gmail.com (Z.N.); wupolalala@gmail.com (Y.S.); 2 Vegetable Research Institute, Hangzhou Academy of Agricultural Sciences, Hangzhou 310024, China; zqianglee@gmail.com (H.W.); luoyeguigen2@yeah.net (J.C.); 3 School of Agriculture and Biology, Shanghai Jiao Tong University, Shanghai 200240, China

**Keywords:** pepper, cytoplasmic male sterility, *restorer-of-fertility* gene, fine mapping

## Abstract

Cytoplasmic male sterility (CMS) is a common biological phenomenon used in hybrid production of peppers (*Capsicum annuum* L.). Although several *restorer-of-fertility* (*Rf*) genes of pepper CMS lines have been mapped, there is no report that the *Rf* gene with clear gene function has been isolated. Here, pepper CMS line HZ1A and its restorer line HZ1C were used to construct (HZ1A × HZ1C) F_2_ populations and map the *Rf* gene. A single dominant gene *CaRfHZ* conferred male fertility according to inheritance analysis. Using sterile plants from (HZ1A × HZ1C) F_2_ populations and bulked segregant analysis (BSA), the *CaRf_HZ_* gene was mapped between P06gInDel-66 and P06gInDel-89 on chromosome 6. This region spans 533.81 kb, where four genes are annotated according to Zunla-1 V2.0 gene models. Based on the analysis of genomic DNA sequences, gene expressions, and protein structures, *Capana06g002968* was proposed as the strongest candidate for the *CaRf_HZ_* gene. Our results may help with hybrid pepper breeding and to elucidate the mechanism of male fertility restoration in peppers.

## 1. Introduction

Cytoplasmic male sterility (CMS) is a common biological phenomenon observed in plants, and it is widely used for seed production of hybrid crops. Fertility restoration of CMS is critical in hybrid production, and *restorer-of-fertility* (*Rf*) gene can restore and affect the fertility of hybrids. More than ten *Rf* genes have been isolated and studied in major crops [1] such as rice, maize, rapeseed, wheat, and soybean. Most of the *Rf* genes in crops contain multiple pentatricopeptide repeat (PPR) domains, such as *Rf1* [2], *Rf4* [3], *Rf5* [4], and *Rf6* [5] in rice; *Rf1* [6] and *Rf3* [7] in maize; *Rfp* [8] in rapeseed; *Rfo* [9] in radish; *RFL79* and *RFL29a* [10] in wheat; and *GmPPR576* [11] in soybean. Additionally, certain *Rf* genes do not contain PPR domains, such as *Rf2* [6] and *Rf4* [12] in maize. *Rf2* is similar to the mammalian aldehyde dehydrogenase, and *Rf4* is a transcription factor that contains basic helix–loop–helix (bHLH) domains. Most *Rf* genes restore the fertility of sterile lines by affecting the transcription of sterile genes in mitochondria. For example, the rice gene *Rf4* restores fertility by reducing the transcripts of the sterility gene *orf352* in the cytoplasm of wild-type rice [4,13]; the rice gene *Rf6* reduces the accumulation of the transcript *atp6-orfH79*, thereby restoring fertility [5]; the maize gene *Rf3* inhibits mitochondrial gene *orf77* editing and degradation, and accelerates *orf355* degradation, leading to CMS-S fertility restoration [14]; the rapeseed gene *Rfp* restores fertility of the sterile line by processing the transcript of the mitochondrial sterility gene *atp6-orf224* [8].

Pepper is one of the largest vegetable crops globally. Peterson (1958) [15] was the first to report cytoplasmic–nucleus interaction type male sterility in pepper, following which, breeders bred numerous sterile pepper lines using CMS lines [16]. Due to the different fertility segregating populations used for genetic analysis in different studies, the inheritance pattern of pepper *Rf* gene also has different conclusions. Wang et al. [17] used the male sterile line 77013A to cross 114 doubled haploid lines to identify the fertility of F_1_ offspring, and considered that fertility restoration of pepper CMS was controlled by one major and four minor genes. Wei et al. [18] analyzed the segregation of fertility using an F_2_ population. The fertility restoration of pepper CMS may be conferred by two pairs of additive-dominant epistatic major genes and additive-dominant polygenes. Gulyas et al. [19] and Ye et al. [20] investigated the fertility separation of F_1_–F_4_ and F_2_ populations constructed using male sterile and restorer lines, respectively. The results showed that a pair of dominant nuclear genes controls fertility restoration in CMS.

In early studies, owing to the limitations of molecular biology techniques and the absence of a reference genome, the *Rf* genes of pepper were only initially mapped. Zhang et al. [21] used the F_2_ population to locate the major *Rf* gene between two randomly amplified polymorphic DNA (RAPD) markers using bulked segregant analysis (BSA), and the genetic distance was 0.37 centimorgans (cM) and 8.12 cM. Wang et al. [17] mapped a major quantitative trait locus (QTL) of fertility restoration to chromosome 6 and four minor QTLs on chromosomes 1, 2, 3, and 5. Kim et al. [22] used BSA–amplified fragment length polymorphism (AFLP) to segregate populations by fertility, constructed a linkage map of the *Rf* gene, and obtained a cleaved amplified polymorphic sequence (CAPS) marker AFRF8CAPS with a genetic distance of 1.8 cM from the *Rf* gene. Lee et al. [23] used 205 F_2_ individual plants and obtained three CAPS and one sequence characterized amplified region (SCAR) markers that were closely linked to the *Rf* site of pepper CMS. Yang et al. [24] used the F_2_ population to obtain the simple sequence repeat (SSR) molecular marker AF208834 linked to the pepper *Rf* gene, with a genetic distance of 20.8 cM. Jo et al. [25] screened the pepper BAC library with the petunia *Rf* gene, cloned the *Rf* candidate gene *PePPR1*, and found three markers closely linked to the *Rf* gene.

Along with the in-depth research and publication of the pepper reference genome [26,27], the pepper *Rf* gene has been mapped to an adjacent region of chromosome 6 in different reports (Table 1). Jo et al. [28] used BSA–AFLP and comparative genomics to locate the *Rf* gene within the 821 kb segment on chromosome 6 and identified the candidate gene *CaPPR6*. Zhang et al. [29] narrowed down the region of *CaPPR6* to 128.96 Kb using kompetitive allele-specific PCR (KASP) markers. Kang et al. [30] mapped an unstable *Rf* gene, *Rfu,* using the molecular markers from Jo et al. [28] and newly developed CAPS markers. Ye et al. [20] located the *Rf* gene between SSR markers pep43 and pep20 on chromosome 6, with a physical distance of 498.6 kb. Wu et al. [31] performed association analysis on 287 pepper lines using specific-locus amplified fragment sequencing (SLAF-seq) and genome-wide association study (GWAS) and identified two candidate *Rf* genes, *Capana06g002967* and *Capana06g002969*. Cheng et al. [32] used high-density genetic mapping and collinearity analysis to map the *Rf* gene *CaRf* to a 270.10 kb region on chromosome 6, and analyzed the candidate gene *Capana06g003028*. Zhang et al. [33] used high-throughput sequencing combined with BSA to initially map the *Rf* gene in the F_2_ population, then mapped the *Rf* gene to a 148.05 kb segment on chromosome 6, and identified the candidate gene *CaRf032* (*CA00g82510*), which has a base variation in the maintainer line leading to premature termination of transcription of the gene. Wei et al. [34] performed RNA sequencing on fertile and sterile pools constructed from the F_2_ population and found a candidate gene, *NEDD8* (*Capana06g002866*), on chromosome 6. However, to date, there is no report that the *Rf* gene with a clear gene function of the pepper CMS line has been cloned.

In the present study, we constructed an F_2_ segregating population and fine-mapped the *Rf* gene in the pepper line HZ1C. The *Rf* gene was mapped to a 533.81 kb region between two insertion or deletion (InDel) markers on chromosome 6 of the Zunla-1 V2.0 reference genome [26]. According to DNA sequence alignment, gene expression, and protein structure analysis, *Capana06g002968* was proposed as the strongest candidate gene for the *Rf* gene in pepper line HZ1C.

## 2. Results

### 2.1. The Inheritance Analysis of Fertility Restoration in CMS Line HZ1A

HZ1A shows the typical characteristics of male sterility, including no pollen dissemination around its anthers and fewer and shriveled pollen grains [35]. The fertility phenotypes were identified through visible pollen on anthers and Alexander’s staining of pollen. All (HZ1A × HZ1C) F_1_ plants were completely male fertile. The segregation of male-fertile to male-sterile plants in 11 F_2_ populations of different sizes, which had been planted in the autumn and spring of 2020 and 2021, respectively, conformed to a 3:1 segregation ratio under the χ^2^ criterion (*p* > 0.05). Overall, 1658 F_2_ plants were classified as 1277 male-fertile and 381 male-sterile plants, which also conformed to a 3:1 segregation ratio (χ^2^ = 3.50, *p* = 0.06) (Table 2). These results demonstrated that a single dominant gene conferred male fertility in HZ1A, which we designated as *CaRf_HZ_*.

### 2.2. BSA and Genetic Linkage Mapping of the CaRf_HZ_ Gene

**Figure 1:**
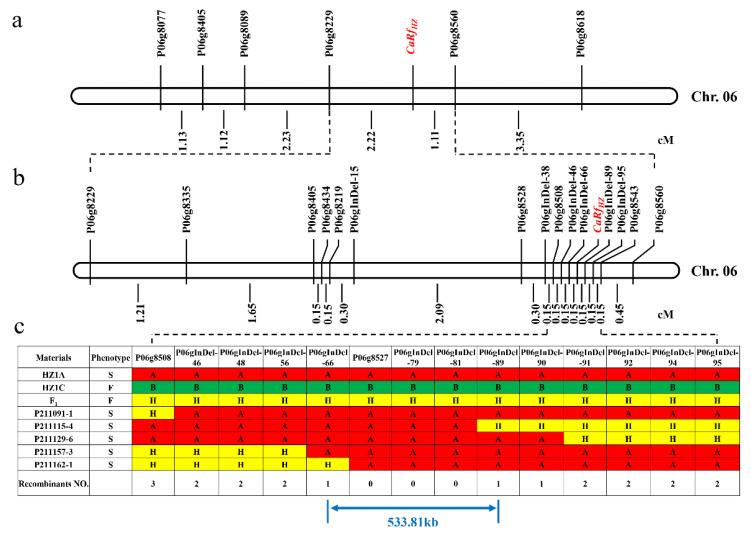
Mapping of the pepper *CaRf_HZ_* gene. (**a**) Primary mapping of *CaRf_HZ_* using a small population of (HZ1A × HZ1C) F_2_. (**b**) Fine mapping of *CaRf_HZ_* using a large population of (HZ1A × HZ1C) F_2_. (**c**) Genotyping results from 5 F_2_ recombinants by 2 SSR markers and 12 newly developed InDel markers: ‘A’ = ‘HZ1A’ genotype; ‘B’ = ‘HZ1C’ genotype; ‘H’ = (HZ1A × HZ1C) F_1_ genotype. Distances are shown in cM. Due to image size limitations, the markers that co-segregated with the fertility phenotype are not all listed in the mapping linkage (**a**,**b**).

In total, 226 plants of the (HZ1A × HZ1C) F_2_ population, which had been planted in the autumn of 2020, were used to map the *CaRfHZ* gene according to BSA. DNA from 10 male-fertile plants was mixed to composite the male-fertile DNA bulk, and DNA from 10 male-sterile plants was mixed to composite the male-sterile DNA bulk. In total, 449 pairs of SSR markers [36] evenly distributed on pepper chromosomes were selected to detect the parents: CMS line HZ1A and restorer line HZ1C. Polymorphic markers with clear bands were detected in male-fertile and male-sterile DNA bulks. Finally, the SSR marker P06g8490 was found to be polymorphic among the DNA bulks. This implied that the *CaRf_HZ_* gene is close to P06g8490 and is located on chromosome 6. In the 12 Mb region near P06g8490, 11 polymorphic SSR markers among the parents were selected. The above 11 SSR markers and P06g8490 were amplified in 45 male-sterile plants from the (HZ1A × HZ1C) F_2_ population. According to the recombinant number and genetic distance between the *CaRf_HZ_* and its linked markers (Table 3), *CaRf_HZ_* was initially located between the markers P06G8229 and P06G8560 (Figure 1a). In this region, 6 SSR markers, P06g8264, P06g8490, P06g8494, P06g8497, P06g8536, and P06g8527, were co-segregated with *CaRf_HZ_*.

### 2.3. Fine Mapping of the CaRf_HZ_ Gene

A larger population composed of 336 male-sterile plants out of the (HZ1A × HZ1C) F_2_ populations, which had been planted in the spring of 2021, was used to narrow down the region of the *CaRfHZ* gene. More SSR markers and newly developed InDel markers (Table 3) in the primary mapping region were selected for fine mapping. Twenty-eight markers were polymorphic in HZ1A and HZ1C, and were used to map the *CaRf_HZ_* gene. The recombinant numbers and genetic distances between the *CaRf_HZ_* gene and its linked markers are shown in Table 3. Some of these markers identified the same number of recombinant individuals, such as P06gInDel-46, P06gInDel-48, and P06gInDel-56, and these markers had the same genetic distance from the *CaRf_HZ_* gene. However, owing to image size limitations, the markers are not listed in the mapping linkage (Figure 1a,b). The figure shows five F_2_ recombinant individuals between the P06G8508 and P06gInDel-95 markers (Figure 1c). Finally, the *CaRf_HZ_* gene was mapped between the markers P06gInDel-66 and P06gInDel-89 with the same genetic distance of 0.15 cM (Figure 1b,c). Three markers, P06G8527, P06gInDel-79, and P06gInDel-81, were co-segregated with the *CaRf_HZ_* gene.

### 2.4. Analysis of the Annotation Genes

According to the pepper reference genome sequence (Zunla-1 V2.0) [26], the physical distance between the two markers, P06gInDel-66 and P06gInDel-89, is 533.81kb on Chr. 06: 215,097,259..215,631,069. In this region, there are four annotated genes (Table 4): *Capana06g002965*, *Capana06g002967*, *Capana06g002968*, and *Capana06g002969* according to Zunla-1 V2.0 gene models.

Full-length genomic DNA sequences of the four annotated genes were sequenced in HZ1A and HZ1C, and the coding sequences were analyzed (Table 5). There were two missense variations at 129 bp and 504 bp and a synonymous variation at 436 bp in *Capana06g002965*. There was a missense variation of 935 bp in *Capana06g002967*. There were two missense variations at 20 bp and 467 bp in *Capana06g002968*. There were two missense variations at 196 bp and 318 bp and a synonymous variation at 144 bp in *Capana06g002969*. Differences in the above sites lead to differences in the amino acid sequence (Figure S1), but only the protein structure of *Capana06g002969* was different. Due to the missense variation at 318 bp, the stop codon TGA of *Capana06g002969* in HZ1C was changed to TGG in HZ1A, which resulted in 31 more amino acids in HZ1A.

**Figure 2:**
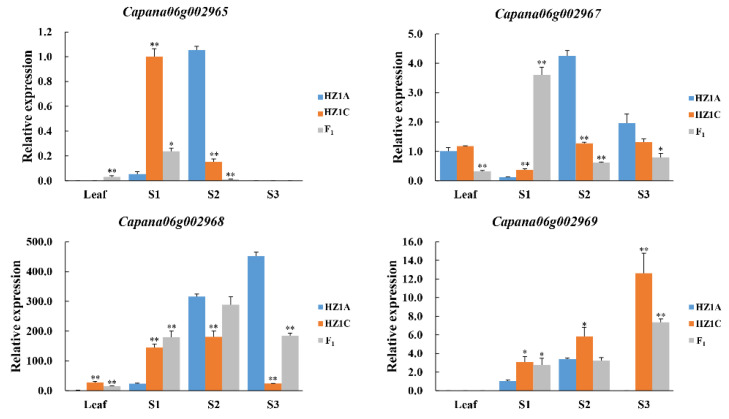
Expression levels of four annotated genes in young leaves and in anthers. * and ** indicate significant difference at *p* < 0.05 and *p* < 0.01, respectively.

The expression of the four annotated genes in young leaves and three developmental stages (stage 1 (S1), sporogenous tissue to meiotic stage; stage 2 (S2), tetrad to mononuclear stage; stage 3 (S3), mature pollen stage) of anthers was analyzed in HZ1A, HZ1C, and (HZ1A × HZ1C) F_1_ using quantitative real-time PCR (qRT-PCR) (Figure 2). The expression of *Capana06g002965* in (HZ1A × HZ1C) F_1_ was low in young leaves but was not detected in HZ1A and HZ1C. At the S1 stage, the expression level of *Capana06g002965* in HZ1A was significantly lower than that in HZ1C and (HZ1A × HZ1C) F_1_. Conversely, the expression level of *Capana06g002965* in HZ1A was significantly higher than that in HZ1C and (HZ1A × HZ1C) F_1_ at the S2 stage. The expression of *Capana06g002965* was not detected in HZ1A, HZ1C, or (HZ1A × HZ1C) F_1_. The expression of *Capana06g002967* in HZ1A was not significantly different from that in HZ1C, but was higher than that in (HZ1A × HZ1C) F_1_ in young leaves and the S3 stage. The expression trends of *Capana06g002967* were similar to *Capana06g002965* at the S1 and S2 stages. The expression level of *Capana06g002968* in HZ1A was significantly lower than that in HZ1C and (HZ1A × HZ1C) F_1_ in young leaves and the S1 stage. At the S2 and S3 stages, the expression levels of *Capana06g002968* in HZ1A were significantly higher than those in HZ1C. The expression of *Capana06g002969* was not detected in the young leaves of HZ1A, HZ1C, and (HZ1A × HZ1C) F_1_. At the S1, S2, and S3 stages, the expression level of *Capana06g002969* in HZ1A was significantly lower than that in HZ1C. In summary, in HZ1A and HZ1C, four genes were differentially expressed at the S1, S2, and S3 developmental stages of anther, with the exception of *Capana06g002967* at the S3 stage. The gene expression trends in HZ1C and (HZ1A × HZ1C) F_1_ were mostly the same.

Although there were differences in the amino acid sequences and gene expression levels of the four annotated genes, it was not possible to determine which gene was the candidate gene for *CaRf_HZ_* based on these differences.

## 3. Discussion

### 3.1. Comparison of CaRf_HZ_ Mapping Interval and the Published Rf Gene Position in Pepper

Thus far, the seven pepper *Rf* genes have been fine mapped using a forward genetics strategy (Table 1). According to the gene mapping intervals, these seven genes are located in an adjacent region of approximately 7 Mb from 210–217 Mb on chromosome 6, and the mapping results overlapped in some studies [20,31]. The situation in which the mapping results are adjacent is presumed to be related to the similarity of the materials used for mapping or to the possible clustering of pepper *Rf* genes on chromosome 6. In the present study, the *CaRf_HZ_* gene was fine-mapped within a 533.81 kb segment in the adjacent region. The mapping interval of the *CaRf_HZ_* gene partially overlaps the mapping region in *Rf* [20] and is located within the mapping range of *Rf* (858.26 kb) [31] and *NEDD8* (5.11 Mb) [34], which are related to the larger mapping range of the above research results. Similar mapping results proved the accuracy of our results in this study but were not conducive to the discovery of new pepper *Rf* genes. As pepper *Rf* genes may exist in clusters on chromosome 6, there may be multiple genes controlling fertility restoration simultaneously. This leads to inconsistent genotypes for the molecular marker *Rf,* making it difficult to locate the *Rf* gene within a shorter interval.

### 3.2. Prediction and Characteristics of the Candidate Gene

According to the annotation information of the Zunla-1 V2.0 genome [26], there are four annotated genes in the localization interval of the *CaRfHZ* gene. All four annotated genes showed sense variations and differences in expression between HZ1A and HZ1C. The candidate gene for *CaRf_HZ_* could not be determined based on the above information.

Therefore, variations between HZ1A and HZ1C were detected in nine maintainer and four restorer lines of HZ1A (Table 5), and missense variations were analyzed. In *Capana06g002965*, missense variations were the same as HZ1A in eight maintainer lines, but they were different from HZ1C in all four restorer lines, and the correlation between missense variations and restored traits was 61.54% in 13 lines. In *Capana06g002967*, missense variations were the same as HZ1C in all four restorer lines, but they were different from HZ1A in eight maintainer lines, and the correlation between missense variations and restored traits was 38.46% in 13 lines. The detection results of missense variations in *Capana06g002968* and *Capana06g002969* were similar; the missense variations were the same as HZ1A in eight maintainer lines and were the same as HZ1C in three restorer lines, and the correlation between missense variations and restored traits was 84.62% in 13 lines. Conclusively, the missense variations in *Capana06g002965* and *Capana06g002967* were not highly correlated with restored traits, while the missense variations in *Capana06g002968* and *Capana06g002969* were highly correlated with restored traits and may be candidate genes for *CaRf_HZ_*. *Capana06g002968* was not reported as a candidate gene for pepper *Rf* in previous studies, but *Capana06g002969* was identified as a candidate gene. There was no difference in *Capana06g002969* sequence between the CMS and restorer lines in a study by Wu et al. [31], which is different from the findings in the present study.

Using the simple modular architecture research tool (SMART) (https://smart.embl.de/, accessed on 15 March 2022) [37] to analyze protein structures, it was found that *Capana06g002968* in HZ1A and HZ1C both contained tetratricopeptide repeat (TPR) domains, and *Capana06g002969* in HZ1A, but not HZ1C, contained an IBR domain. No reports related to plant fertility were found for the IBR domain, but there have been reports on the TPR domain. In rice, silencing the gene *ORF3* containing the TPR domain can restore the spikelet fertility of heterozygous plants [38]. The C-terminal TPR and CaMbd domains of the *wFKBP73* gene were critical for male fertility in transgenic rice [39]. Overexpression of *AtTRP1*, which contains a TRP domain, in wild-type *Arabidopsis* results in dwarf plants and reduced fertility [40]. High expression of TPR may affect normal plant fertility. In our study, anther development of HZ1A started abnormally from the S2 stage, and the expression of *Capana06g002968* in HZ1C was lower than that in HZ1A in the S2 and S3 stages. This suggests that lower expression of *Capana06g002968* may be involved in the restoration of fertility of HZ1A. Therefore, *Capana06g002968* was proposed as the strongest candidate gene for *CaRf_HZ_*, but this needs to be validated in the future.

### 3.3. Application of the Related Markers of CaRf_HZ_ Gene in Pepper Breeding

Since the discovery of the first pepper CMS by Peterson [15], CMS sterile lines have been used for pepper breeding. The ‘three-line’ hybrid seed production system consisting of CMS, maintainer, and restorer lines is used in pepper seed production. This method can reduce the link of manual emasculation, thereby reducing the production cost and improving the purity of the hybrid. Several excellent pepper hybrid varieties were obtained by this method in China [41,42]. Fertility stability of the hybrid is an important guarantee for the large-scale promotion of the ‘three-line’ hybrid, and the *Rf* gene in the restorer line plays a key role.

Based on the differential variations in *Capana06g002968* (467 bp) and *Capana06g002969* (318 bp) in HZ1A and HZ1C, we developed two CAPS markers (results not shown). Amplification and enzyme digestion were performed on 13 lines (Table 5), and the results were consistent with the sequencing results. The accuracy of detecting both maintainer and restorer lines was 84.62% (*Capana06g002968*) and 92.31% (*Capana06g002969*), but not 100%, which may be related to the existence of other fertility restorer genes. The accuracies of 84.62% and 92.31% were similar to those of previous studies, such as Co1Mod1-CAPS (88.0% and 92.2%, respectively) [28], and CRF3S1S, CRF-SCAR, and Co1Mod1-CAPS markers (77.2%, 79.2%, and 70.3%, respectively) [33]. The newly developed CAPS markers in this study can be used for sterile and restorer line screening, but they need validation in more maintainer and restorer lines.

## 4. Materials and Methods

### 4.1. Plant Materials

The CMS line HZ1A is a pepper line with complete sterility that is obtained by backcrossing for more than 15 generations. The F_2_ segregating populations (Table 2) were derived from 11 F_1_ hybrid plants, with HZ1A and HZIC as the female and male parents, respectively. In total, 226 F_2_ plants and 1442 F_2_ plants were planted in the autumn of 2020 and spring of 2021, respectively. Plants of F_2_ populations were used for inheritance analysis and mapping of the *Rf* gene *CaRf_HZ_*. All plant materials, consisting of HZ1A, HZ1C, maintainer lines, restorer lines, F_1_ plants, and F_2_ plants, were grown in a plastic-covered greenhouse at the Zhuanghang Experimental Station of Shanghai Academy of Agricultural Sciences (Shanghai, China).

### 4.2. Pollen Fertility Evaluation

Pollen fertility was evaluated visually with at least three blooming flowers and further discriminated using Alexander’s solution staining [43] under microscope, as described previously [35]. Plants that produced abundant pollen grains were considered male-fertile, whereas plants with no pollen dissemination were considered male-sterile. After Alexander’s solution staining, the pollen grains that stained magenta red were classified as male-fertile, and those stained blue–green were classified as male-sterile.

### 4.3. Inheritance Analysis of Fertility Restoration

Segregation of male fertility/sterility was identified based on the fertility of the plants from the (HZ1A × HZ1C) F_2_ population. Chi-square goodness-of-fit test was performed using the following equation: *χ*^2^ = Σ (*O* − *E*)^2^/*E*, where *O* is the observed frequency, and *E* is the expected frequency, with *p* = 0.05 as the threshold.

### 4.4. Nucleic Acid Extraction

Genomic DNA was extracted from the young leaves using a DNA extraction kit (Tiangen, Beijing, China). The anthers of HZ1A, HZ1C, and (HZ1A × HZ1C) F_1_ at three developmental stages: stage 1 (S1), sporogenous tissue to meiotic stage; stage 2 (S2), tetrad to mononuclear stage; and stage 3 (S3), mature pollen stage, based on an earlier study [35] were isolated from the flower buds and immediately placed into an RNA stabilization solution (Qiagen, Hilden, Germany). Total RNA was isolated from anthers using a Biospin Plant Total RNA Extraction Kit (Bioer Technology, Hangzhou, China). Reverse transcriptase was used to enrich full-length cDNA using a HiScript II One Step RT-PCR Kit (Vazyme, Nanjing, China). According to the manufacturer’s instructions, DNA and RNA were extracted, and reverse transcription was performed.

### 4.5. SSR and InDel Markers

SSR markers used in the present study were derived from Cheng et al. [36]. SSR markers are numbered according to their position on the chromosome, and chromosome information is added, such as P01g0001. The markers used in the *CaRf_HZ_* linkage map are shown in Table S1.

Chromosome 6 sequences of two published pepper genomes, Zunla-1 V2.0 and Chiltepin V2.0, were obtained from the China National GeneBank Sequence Archive (CNSA) (https://db.cngb.org/search/project/CNPhis0000547/, accessed on 25 October 2020). According to the initial and fine mapping of the *CaRf_HZ_* gene, 4 Mb (Chr06:195,000,000..196,000,000 and Chr06: 213,000,000..216,000,000) sequences were selected to search for InDel. The InDel variations between ‘Zunla-1’ and ‘Chiltepin’ were detected using MUMmer 3.0 software [44] (Hamburg, Germany), and the parameter of variation length ≥ 5 bp was used for screening. The 500 bp sequences before and after the InDel variation were selected, and Primer-BLAST software (Bethesda, MD, USA) was used for primer sequence design. In total, 102 InDel markers were developed, and the information is listed in Table S2. Sangon Biotech (Shanghai, China) synthesized the primers. The design and synthesis of all primers used in the present study were the same as those described above.

### 4.6. BSA and Linkage Analysis of CaRf_HZ_ Gene

The *CaRf_HZ_* gene was mapped using BSA. DNA from 10 plants each from male-fertile and male-sterile plants of the (HZ1A × HZ1C) F_2_ population were randomly selected and mixed to composite the male-fertile and male-sterile DNA bulks. Then, 449 SSR markers [36], which covered the entire pepper genome, were tested for polymorphism in the parent HZ1A and HZ1C. Thereafter, the polymorphic markers between parents were detected in male-fertile and male-sterile DNA bulks. The polymorphic marker between DNA bulks was recognized as linked to the *CaRf_HZ_* gene.

In the present study, the male-sterile plants from the (HZ1A × HZ1C) F_2_ populations were used to map the *CaRf_HZ_* gene (Table 2) according to Zhang et al. [45]. The polymorphic markers between DNA bulks and the SSR and InDel markers near this marker were amplified and analyzed in male-sterile plants from the (HZ1A × HZ1C) F_2_ population. The recombination rate (c) between the marker and the *CaRf_HZ_* gene was calculated using the formula *c* = (*N*1 + *N*2/2)/*N*, where *N* is the number of all sterile plants, *N*1 is the number of plants with homozygous bands from the fertile parent HZ1C, and *N*2 is the number of plants with heterozygous bands from the two parents [45,46]. Kosambi’s formula: *d* = 1/4 log ((1 + 2*c*)/(1 − 2*c*)) was used to calculate the genetic distance (*d*) [47]. The unit of *d* is centimorgans (cM). MapDraw v2.1 was used to construct the genetic map [48].

PCR amplification was performed using a reaction mixture containing the following: 5 µL of 2×Hieff^®^ PCR Master Mix (YEASEN, Shanghai, China), 1 μL of 10 µM PCR upstream primer, 1 μL of 10 µM PCR downstream primer, 20 ng template DNA, and ddH_2_O up to 10 µL. The PCR cycling program was as follows: pre-denaturation at 94 °C for 2 min; followed by 32 cycles of 94 °C for 30 s, primer annealing temperature for 30 s, and 72 °C for 30 s; final extension at 72 °C for 5 min; cooling to 12 °C. Amplification products were electrophoresed using 8% non-denaturing polyacrylamide gels at 150 V for 1 h. The gels were visualized using 0.1% AgNO_3_ solution [46].

### 4.7. Candidate Gene Amplification and qRT-PCR

Amplification of the candidate genes of *CaRf_HZ_* in the HZ1A and HZ1B parental lines was performed following the instructions using GoldenStar^®^ T6 Super PCR Mix Ver.2 (Tsingke Biotechnology, Beijing, China) in a total reaction volume of 50 µL containing 45 μL of 1.1× GoldenStar^®^ Mix Ver.2, 2 μL of 10 µM upstream primer, 2 μL of 10 µM downstream primer, and 200 ng template DNA. The PCR cycling conditions were as follows: pre-denaturation at 98 °C for 2 min; followed by 32 cycles of 98 °C for 10 s, primer annealing temperature for 15 s, and 72 °C for 40 s; final extension at 72 °C for 5 min, and cooling to 12 °C. The primers used for cloning the candidate genes of *CaRf_HZ_* are listed in Table S3. PCR products were sequenced by Sangon Biotech (Shanghai, China). The amino acid sequences of the candidate genes of *CaRf_HZ_* were aligned using BioXM 2.7.120 (Nanjing, China).

The qRT-PCR was performed using 2x Hieff UNICON^®^ Universal Blue qPCR SYBR Green Master Mix (YEASEN, Shanghai, China) following the instructions. The PCR reactions were analyzed using the Quant Studio 5 real-time PCR system (Waltham, MA, USA). The *β-actin* gene from Lv et al. [49] was used as an internal control to normalize expression data. The 2^−ΔΔCt^ method was used to calculate the relative expression levels. Three biological and three technical replicates were used for each experiment. The primers used for qRT-PCR are listed in Table S4.

### 4.8. Analysis of Candidate Genes

The physical positions of the molecular markers were determined with reference to the pepper reference Zunla-1 V2.0 genome sequence as described above. The annotated genes in the identified target region were obtained according to the Zunla-1 V2.0 gene models [26]. The protein structures of annotated genes were predicted by SMART (https://smart.embl.de/, accessed on 15 March 2022) [37].

## 5. Conclusions

To summarize, the male fertility of the pepper CMS line HZ1A could be restored by a single dominant gene, *CaRf_HZ_*. The *Rf* gene, *CaRf_HZ_*, was fine-mapped to a 533.81 kb region on chromosome 6 using BSA. Four annotated genes were analyzed according to Zunla-1 V2.0 gene models. Based on the comparison of genomic DNA sequences, qRT-PCR, and protein structures in HZ1A and its maintainer and restorer lines, *Capana06g002968* was proposed as the strongest candidate gene for the *CaRf_HZ_* gene. Functional verification of the *CaRfHZ* gene needs to be performed in the future.

## Figures and Tables

**Table 1:** Reported *Rf* candidate genes on chromosome 6 in pepper.

Gene Name	Mapping Method	Range	Flanking Markers	Mapping Interval (Zunla-1 V2.0)	Candidate Genes	Locations of Candidate Genes (Zunla-1 V2.0)	References
*Rf*	CAPS, SSR, SCAR markers and BSA	498.60 kb	pep20~pep43	Chr06: 214,671,854.. 215,170,454	*Capana06g002962* *Capana06g002963* *Capana06g002965*	Chr06: 214,791,005..214,791,707 Chr06: 214,928,883..214,932,088 Chr06: 215,102,194..215,102,754	[20]
*CaPPR6*	BSA–AFLP and comparative mapping	128.96 kb	S423~S424	Nearby Chr06: 216,988,988	*CaPPR6*	Chr06: 214,033,734..214,035,560	[28,29]
*Rfu*	CAPS, SCAR markers	398.00 kb	4162-SCAR ~G16-CAPS	Nearby Chr06: 214,076,189	*CA00g30080*	-	[30]
*Rf*	SLAF-seq, and GWAS	858.26 kb	P06-247 ~PW6-126	Chr06: 214,868,888.. 215,727,145	*Capana06g002967* *Capana06g002969*	Chr06: 215,172,352..215,173,332 Chr06: 215,340,394..215,340,521	[31]
*CaRf*	Conjoint analysis of recombinants and collinearity	270.10 kb	S3~S1	Nearby Chr06: 217,065,420	*Capana06g003028*	Chr06: 217,273,835..217,280,134	[32]
*CaRf032*	Genome resequencing and recombination analysis	148.05 kb	S1402~S1354	Chr06: 213,923,525.. 214,071,576	*CA00g82510*	Chr06: 214,033,838..214,035,589	[33]
*NEDD8*	Bulked segregant RNA sequencing	5.1 Mb	KS18~KS22	Chr06: 210,576,870.. 215,685,280	*Capana06g002866*	Chr06: 210,452,333..210,452,887	[34]

“-” indicates there is no corresponding region. In some studies, CM334 v1.55 was selected as the reference genome; therefore, the flanking markers of the mapping interval were compared on the Zunla-1 V2.0 genome to obtain the corresponding positions. For the markers without corresponding positions, according to the corresponding positions of the annotated genes in the mapping interval on the Zunla-1 V2.0 genome, the approximate locations of the mapping intervals, such as *CaPPR6* [28,29], *Rfu* [30], and *CaRf* [32] were speculated. The mapping interval of *NEDD8* [34] in the original text is inconsistent with the candidate gene position.

**Table 2:** Segregation patterns of male fertility and sterility in the pepper F_2_ population derived from the cross HZ1A × HZ1C.

Year and Season	Line Name	No. of Plants			Expected Ratio	*χ* ^2^	Probability
		Total	Fertile	Sterile			
2020 Autumn	P20210	66	53	13	3:1	0.73	0.39
	P20217	63	53	10		2.33	0.13
	P20218	97	65	22		0.00	0.95
2021 Spring	P211014	134	102	32	3:1	0.04	0.84
	P211017	370	279	91		0.01	0.90
	P211091	129	102	27		0.93	0.33
	P211103	168	134	34		1.79	0.18
	P211115	160	128	32		1.88	0.17
	P211129	161	121	40		0.00	0.96
	P211157	218	165	53		0.02	0.88
	P211162	102	75	27		0.05	0.82
Total		1658	1277	381	3:1	3.50	0.06

**Table 3:** The recombinant number and genetic distance between the *CaRf_HZ_* gene and its linked markers.

	Marker	Position (bp)	Recombinant Individuals	Genetic Distance (cM)
Primary mapping	P06g8405	203,656,182	3 HO	6.71
	P06g8077	212,732,221	2 HO, 1 HE	5.58
	P06g8089	204,569,609	2 HO	4.46
	P06g8229	208,899,271	2 HE	2.22
	*CaRf_HZ_*			
	P06g8264	209,767,667	0	0.00
	P06g8490	214,353,887	0	0.00
	P06g8494	214,428,701	0	0.00
	P06g8497	214,494,826	0	0.00
	P06g8536	215,672,797	0	0.00
	P06g8527	215,336,423	0	0.00
	P06g8560	215,995,316	1 HE	1.11
	P06g8618	216,556,879	2 HO	4.46
Fine mapping	P06g8229	208,899,271	43 HE	6.43
	P06g8335	210,959,629	35 HE	5.23
	P06g8405	212,732,234	24 HE	3.58
	P06g8434	213,257,869	23 HE	3.43
	P06g8219	208,736,376	22 HE	3.28
	P06gInDel-15	213,906,630	20 HE	2.98
	P06g8528	215,338,579	6 HE	0.89
	P06gInDel-38	214,431,109	4 HE	0.60
	P06g8490	214,353,887	4 HE	0.60
	P06g8499	214,498,896	4 HE	0.60
	P06g8508	214,872,305	3 HE	0.45
	P06gInDel-46	214,885,975	2 HE	0.30
	P06gInDel-48	214,903,798	2 HE	0.30
	P06gInDel-56	214,950,959	2 HE	0.30
	P06gInDel-66	215,097,259	1 HE	0.15
	*CaRf_HZ_*			
	P06g8527	215,336,423	0	0.00
	P06gInDel-79	215,419,831	0	0.00
	P06gInDel-81	215,498,386	0	0.00
	P06gInDel-89	215,631,069	1 HE	0.15
	P06gInDel-90	215,633,948	1 HE	0.15
	P06gInDel-91	215,636,889	2 HE	0.30
	P06gInDel-92	215,669,032	2 HE	0.30
	P06gInDel-94	215,669,384	2 HE	0.30
	P06gInDel-95	215,674,072	2 HE	0.30
	P06g8543	215,710,422	3 HE	0.45
	P06gInDel-99	215,741,617	3 HE	0.45
	P06g8549	215,773,438	3 HE	0.45
	P06g8560	215,995,331	6 HE	0.89

‘HO’ indicates a recombinant individual with a homozygous band from the fertile parent HZ1C, and ‘HE’ indicates a recombinant individual with heterozygous bands from the two parents.

**Table 4:** List of four annotation genes in the mapping region of *CaRf_HZ_*.

Gene ID	Position (bp)	Annotation Description
*Capana06g002965*	Chr06:215,102,194..215,102,754	Unknown protein
*Capana06g002967*	Chr06:215,172,352..215,173,332	CW-type zinc finger protein
*Capana06g002968*	Chr06:215,328,827..215,334,970	Tetratricopeptide repeat protein
*Capana06g002969*	Chr06:215,340,394..215,341,013	Unknown protein

**Table 5:** The variations of four annotated genes in the CMS, maintainer, and restorer lines.

Line name	Type	*Capana06g002965*			*Capana06g002967*	*Capana06g002968*		*Capana06g002969*		
		129 bp	436 bp	504 bp	935 bp	20 bp	467 bp	144 bp	196 bp	318 bp
HZ1A	CMS line	A	T	G	G	G	T	G	T	G
HZ1C	Restorer line	C	C	A	A	C	G	A	G	A
P21204	Maintainer line	C	T	G	G	G	G	G	G	G
P21238	Maintainer line	A	T	G	A	G	T	A	T	G
P21239	Maintainer line	A	T	G	A	G	T	A	T	G
P21241	Maintainer line	A	T	G	A	G	T	A	T	G
P21243	Maintainer line	A	T	G	A	G	T	A	T	G
P21244	Maintainer line	A	T	G	A	G	T	A	T	G
P21246	Maintainer line	A	T	G	A	G	T	A	T	G
P21270	Maintainer line	A	T	G	A	G	T	A	T	G
P21273	Maintainer line	A	T	G	A	G	T	A	T	G
P21215	Restorer line	A	T	G	A	C	G	A	G	A
P21240	Restorer line	A	T	G	A	C	G	A	G	A
P21247	Restorer line	A	T	G	A	C	G	A	G	A
P21274	Restorer line	A	T	G	A	G	T	A	T	G

Shading indicates the nucleobases are consistent with the restored traits.

## Data Availability

Not applicable.

## Supplementary Materials

The following supporting information can be downloaded at: https://www.mdpi.com/article/10.3390/ijms23147633/s1.
