# Supplementary material for: Fine Mapping and Gene Analysis of restorer-of-fertility Gene CaRfHZ in Pepper (Capsicum annuum L.)
Source: Int J Mol Sci. 2022 Jul 11;23(14):7633. doi: 10.3390/ijms23147633 (PMC9316182; doi:10.3390/ijms23147633)
Supplement: Supplementary file 1 [file ijms-23-07633-s001.zip › Figure S1.pdf]

>Capana06g002965

```
HZ1A     MADQENPLTTMKKTLFYKFYPSGDEEEACKAKNVPWLVRRELIEIRDTYPVPIIDLENPWKFKKKIDEHDFTQTGLLIPFLVAFEHIIRYWKLPDGKTLV 100
HZ1C     MADQENPLTTMKKTLFYKFYPSGDEEEACKAKNVPWLVRRELIEIRDTYPVPIIDLENPWKFKKKIDEYDFTQTGLLIPFLVAFEHIIRYWKLPDGKTLV 100
Zunla-1  MADQENPLTTMKKTLFYKFYPSGDEEEACKAKNVPWLVRRELIEIRDTYPVPIIDLENPWKFKKKIDEYDFTQTGLLIPFLVAFEHIIRYWKLPDGKTLV 100

HZ1A     NGNWWNVVLCDVTEENDLKKYDGASGRSFRFGKMWDDDYFLQCRQLFIDRGLGVGDEIGLYWDPWSSMFMFKLLSKFGDSRIRTKH 186
HZ1C     NGNWWNVVLCDVTEENDLKKYDGASGRSFRFGKMWDDDYFLQCRQLFIDRGLGVGDEIGLYWDPWSSIFMFKLLSKFGDSRIRTKH 186
Zunla-1  NGNWWNVVLCDVTEENDLKKYDGASGRSFRFGKMWDDDYFLQCRQLFIDRGLGVGDEIGLYWDPWSSIFMFKLLSKFGDSRIRTKH 186
```

>Capana06g002967

```
HZ1A     MAEEVEVEEGEIFQDYCADIDMKIERCLGEYRKEFEGIVSFENLGPRFGMYGSFLPCYQRPPSLLFHPTIQGVPSASQFSSQQSNSIELNHEATTSSIGG 100
HZ1C     MAEEVEVEEGEIFQDYCADIDMKIERCLGEYRKEFEGIVSFENLGPRFGMYGSFLPCYQRPPSLLFHPTIQGVPSASQFSSQQSNSIELNHEATTSSIGG 100
Zunla-1  MAEEVEVEEGEIFQDYCADIDMKIERCLGEYRKEFEGIVSFENLGPRFGMYGSFLPCYQRPPSLLFHPTIQGVPSASQFSSQQSNSIELNHEATTSSIGG 100

HZ1A     PVSLRKKVVKRVASHRDEPPVSPFIIQGDWICCVHCCKWRLLPYGTKREQLSDSWLCSMLDWLPGMDHCDFTEEDTTRGLHSIYQSLILNNFQNRDGKGS 200
HZ1C     PVSLRKKVVKRVASHRDEPPVSPFIIQGDWICCVHCCKWRLLPYGTKREQLSDSWLCSMLDWLPGMDHCDFTEEDTTRGLHSIYQSLILNNFQNRDGKGS 200
Zunla-1  PVSLRKKVVKRVASHRDEPPVSPFIIQGDWICCVHCCKWRLLPYGTKREQLSDSWLCSMLDWLPGMDHCDFTEEDTTRGLHSIYQSLILNNFQNRDGKGS 200

HZ1A     IAHNGREISVKKRKLKDQDCLVTLRCNGNDLGDGDINAVDREVSRAFRKQKISKRESSTRDTVARIGIKDSPPTDKSAAEREHQTKKYRVGHQSQAEFGI 300
HZ1C     IAHNGREISVKKRKLKDQDCLVTLRCNGNDLGDGDINAVDREVSRAFRKQKISKRESSTRDTVARIGIKDSPPTDKSAAEREHQTKKYRVGHQSQAEFGI 300
Zunla-1  IAHNGREISVKKRKLKDQDCLVTLRCNGNDLGDGDINAVDREVSRAFRKQKISKRESSTRDTVARIGIKDSPPTDKSAAEREHQTKKYRVGHQSQAEFGI 300

HZ1A     IRKQVCKFPRKRALGYGKEARTCIAI 326
HZ1C     IRKQVCKFPRKQALGYGKEARTCIAI 326
Zunla-1  IRKQVCKFPRKQALGYGKEARTCIAI 326
```

>Capana06g002968

```
HZ1A     MEMEVVSTKEKKEKEQFHVFHKVPSGDGPYVRAKHVQLILKDPEGSIVWFWKAINEGDRVDSALKDMAVVMKQLDRSEEAIEAVKSFRYLCSKQAQESLD 100
HZ1C     MEMEVVTTKEKKEKEQFHVFHKVPSGDGPYVRAKHVQLILKDPEGSIVWFWKAINEGDRVDSALKDMAVVMKQLDRSEEAIEAVKSFRYLCSKQAQESLD 100
Zunla-1  MEMEVVTTKEKKEKEQFHVFHKVPSGDGPYVRAKHVQLILKDPEGSIVWFWKAINEGDRVDSALKDMAVVMKQLDRSEEAIEAVKSFRYLCSKQAQESLD 100

HZ1A     NVLFDLFKKCGKVEEQILLLKHKLRQIYEGKLFNGRPVKVARSHGKKIQVTISQETSRVLGNLGWAYMQKGNFMAAEVVYKKAQMIYPDSNKACNLAHCL 200
HZ1C     NVLFDLFKKCGKVEEQILLLKHKLRQIYEGKLFNGRPVKVARSHGKKIQVTISQETARVLGNLGWAYMQKGNFMAAEVVYKKAQMIYPDSNKACNLAHCL 200
Zunla-1  NVLFDLFKKCGKVEEQILLLKHKLRQIYEGKLFNGRPVKVARSHGKKIQVTISQETARVLGNLGWAYMQKGNFMAAEVVYKKAQMIYPDSNKACNLAHCL 200

HZ1A     IKQARYDEARSILDDVWRGKYLGSDDKKTKNRVEELLLELDSKQPPPFLQNIPGLDLDDDFVNGLEQLIHEWARPKSRRLPIFEEISTFRDQLAC 295
HZ1C     IKQARYDEARSILDDVWRGKYLGSDDKKTKNRVEELLLELDSKQPPPFLQNIPGLDLDDDFVNGLEQLIHEWARPKSRRLPIFEEISTFRDQLAC 295
Zunla-1  IKQARYDEARSILDDVWRGKYLGSDDKKTKNRVEELLLELDSKQPPPFLQNIPGLDLDDDFVNGLEQLIHEWARPKSRRLPIFEEISTFRDQLAC 295
```

>Capana06g002969

```
HZ1A     MDPRDIQIFNRDEKTDSQLSFDINENEEGIMDEDNDIELQQILFYSAQFHSGKNSESRSSNAAENSNREAKVLASPYIVDCPYVDCTGKLVDDGKEYLIR 100
HZ1C     MDPRDIQIFNRDEKTDSQLSFDINENEEGIMDEDNDIELQQILFYSAQFHSGKNSESRSSNAAENANREAKVLASPYIVDCPYVDCTGKLVDDGKEYLIR 100
Zunla-1  MDPRDIQIFNRDEKTDSQLSFDINENEEGIMDEDNDIELQQILFYSAQFHSGKNSESRSSNAAENANREAKVLASPYIVDCPYVDCTGKLVDDGKEYLIR 100

HZ1A     TCPNFWRIFCVRCRNAWHCGITFEAYKHMLGQMNFFV 137
HZ1C     TCPNF-------------------------------- 105
Zunla-1  TCPNF-------------------------------- 105
```
