# Supplementary material for: Fine Mapping and Gene Analysis of restorer-of-fertility Gene CaRfHZ in Pepper (Capsicum annuum L.)
Source: Int J Mol Sci. 2022 Jul 11;23(14):7633. doi: 10.3390/ijms23147633 (PMC9316182; doi:10.3390/ijms23147633)
Supplement: Supplementary file 1 [file ijms-23-07633-s001.zip › Supplementary Tables.pdf]

Table S1. Primer sequences of SSR markers used for mapping *CaRf*$_{HZ}$.

Name	Upstream primer (5′-3′)	Downstream primer (5′-3′)	Position (bp)	Size (bp)
P06g8077	TACCTGGATAGTGGATGGCC	ACTTGTGGGTGGTCTTGGAA	203,656,182	350
P06g8089	AGAAAACGGAGAGAGCAAAACT	GGATTTACTCTGCATGACGGG	204,569,609	359
P06g8219	TGGTGGATGCTTAAGGTCGT	CCTTCGCACTGAGTACATTGT	208,736,376	478
P06g8229	TGAAACCCCACTTCCTCCTC	AGGCATTACACCACTCACCA	208,899,271	264
P06g8264	ACCCTTCCCAAATCCCACTT	AACGAAATACCGGGTTGTGC	209,767,667	250
P06g8335	ACCCCTCCCAAGAAGCTTTT	CACACGAACTGACCGAACTC	210,959,612	413
P06g8405	ACCACAAAACACACACACAGT	TCCCAAAGTTACTGCACTGC	212,732,221	489
P06g8434	TGGTTGTTGATGGTTGCTGG	CAACAACAACAGCAACCCCT	213,257,858	188
P06g8490	ACCATTCGTTGTATCTTTGCCT	ACGAGAGGGTCCATTGCTTT	214,353,887	311
P06g8494	GAGAAGAAAAGTGGGCGGTC	TCAAACTCAAAGCCCGTGTG	214,428,701	428
P06g8497	TGCAGTAGGAGTGAGTACGG	ACCCTTTGATGATGAATGTGCT	214,494,826	496
P06g8499	ACAAGGTCGAGAGGAGATCA	TGCATGGAGTTGGATATGTGA	214,498,896	417
P06g8508	ACGTTGCTAGGAGAAATTAAGAGT	TCGAACCAACTGATAAACCGA	214,872,261	299
P06g8527	GCTTGTTTCCAACTTCATCCC	TGGGTGCCAAATCTGAAAACA	215,336,423	319
P06g8528	ACATGAAAGCTGCACAATTCCT	ACTCAAAATGACGCGTGTGG	215,338,579	337
P06g8536	AGCAGACAAGAAAAGCAGGC	CGTCAAAGGTGTCACGTCAG	215,672,797	282
P06g8543	GCACCTGTTAGAAGCAACGG	AGATCATGGCAGCGCTACTA	215,710,422	244
P06g8549	CCGCCAAAATCCTACGCAAT	TTAGCCGCATGTACAGGTGT	215,773,438	469
P06g8560	AAGCATCCAGTACTCCCTCC	CGTTGCCGGTGACATAGTAT	215,995,316	275
P06g8618	TGTTTTGCTTACTTTCTGCTTCT	GAAACCAAGGCTGACTGACA	216,556,879	223

Table S2. Information of InDel markers developed in the present study.

Name	Upstream primer (5′-3′)	Downstream primer (5′-3′)	Position (bp)	Size (bp)
P06gInDel-01	TTTCAGCCATATCCAACTTCTGC	CTCCATCATCTGTCCATTTTTCC	195,010,051	250
P06gInDel-02	CTTGACCACCCCCATCATGT	CAGCCTACTCTCTTTGCCGT	195,014,153	213
P06gInDel-03	TAGCACTAGTGTTGGGAGGC	TCATGGATTCTGCACTGCGT	195,340,084	215
P06gInDel-04	CGGGATAGCTAAACCCAGTGA	CCGGAACCCCATAACACGAT	195,429,448	189
P06gInDel-05	GGACAAATATGCCCCCGAATTATG	TGCGTTAGAGGGCATTTAGC	195,431,220	242
P06gInDel-06	GTCGTGTTGTCGGCTACTTG	ACCAAACGACCCCTAATTGTGT	195,433,716	227
P06gInDel-07	CTGGTACCCCCTCCTGAACT	GGGTTTCAATTACCCCCGGAA	195,672,142	232
P06gInDel-08	AGGTGCGCACAAACAAATCC	CCGACACGGTGCGACAATA	195,732,144	216
P06gInDel-09	TAGGATGCCTCCACCCTACC	GGGTGAAGTGTTTTAAGGGTGTG	195,778,184	200
P06gInDel-10	GGCAAATGATCTTTTCGAATCTCCT	TGAGAGAAATGACTCTCTAACTCGT	195,817,379	138
P06gInDel-11	ATCTTTCACCTGTCATTTGTCTGT	TTGATCGACCTGATTGGCTCT	195,895,444	93
P06gInDel-12	GGCATGTCCCGACTTTGTTG	CACTTTTCTAAGGGCCTTCACAC	195,928,777	210
P06gInDel-13	AGTTCCATAATCGATTTCCCCA	AGGGCATTTACGCCGGTTA	213,856,902	203
P06gInDel-14	CTACCTGTCGATGGAGCGAG	CCAACAGACACCTAGCAGCA	213,870,118	241
P06gInDel-15	AGCTGGCACAGTAACAAGCA	CATGGACATAAATGTCTGAAAACCA	213,906,630	222
P06gInDel-16	ACAAGCATCTCTAATTGGTCTGT	CCAATTGTGGTACCATTAATCAACT	213,907,565	203
P06gInDel-17	ACTTGGTAGGCTGTTGCTCA	GGTCCTGCAGTACGCTATCC	213,911,020	180
P06gInDel-18	ACCTTAGTTGACATCTACGCCC	AACTTGGCCGCGATTGTCTT	213,973,846	239
P06gInDel-19	CCGGAATTACTTTTGAAAGGCCA	AAAAATGCTCCATCAGAGTTTCAA	214,041,961	235
P06gInDel-20	GCAATCACAGACCCTGGACA	TTTCCAACGTCACCTACCGT	214,112,361	145
P06gInDel-21	AGGGCATTGCAACTTCACCA	GGGTCATGTGGTTTGAAGACA	214,114,992	247
P06gInDel-22	AGCGAACATGTAAAATGAAGGATCA	TTGCCAAGAAGTCGATTGGC	214,145,765	213
P06gInDel-23	TCAGCATAGCGGCACTCTTA	CCTTTGTGGGGTAGGGAGGT	214,152,225	203
P06gInDel-24	TTGAACCACTAGGCCACACC	GTTTTTGGTAAGGTAAGCCCCC	214,173,876	153
P06gInDel-25	ACGTAAGCACACGCTTTTAACT	TCGGTTTTGAACCTGTCGAG	214,180,331	235
P06gInDel-26	CGTCATAGATGTGAGTTGAGGC	TGAACCTTGGGAAATTACGTTCA	214,192,496	240
P06gInDel-27	TTTTGCTCTACACCAAACACACC	AGAGATTCTGCAGGAGTTGTCG	214,223,743	183
P06gInDel-28	TCGTATTGTTTTGAGTCGGGGT	ACGCAGTCTGTACCACTATTT	214,285,055	218
P06gInDel-29	TGTGAGTGTGTGACACGGTT	ACACCTCGGAAAGTGAACCA	214,287,980	195
P06gInDel-30	GGGTGGGTATTAACTACTAACACAA	GAGTTGGGAGGACGACCAAG	214,304,572	235
P06gInDel-31	TGAAGGTAGACCTTGATTTGGAT	ACACTCTAACTCTACGGAGGGA	214,331,186	216
P06gInDel-32	ACATGCATGAGGTTAGGTCGT	ACCAGATGGGTTACTCCACA	214,333,763	170
P06gInDel-33	CGAATGCATTACAATAGTTTCGTGT	ACAAGTTGGTGGCAGGGTAG	214,341,579	139
P06gInDel-34	TGATTGATGTTGTAGCACGCC	TGGGTTCTACAGTTCGCGATT	214,367,545	232
P06gInDel-35	CGTTTGCGTTGTTTTCCCCG	GGAGGTCCCATTTGAGTGGT	214,375,839	227
P06gInDel-36	AGGGTCCGAAGGATGTTCTG	ACATGCCCCCGACTATGAC	214,407,581	220
P06gInDel-37	TGAGTGGCCATTCTATTATCTTCT	TCTCGATCAACAATTCTGGCA	214,412,577	249
P06gInDel-38	TTGTGAACAGATAAGGAGAGCTT	GGCAAAAACGTTACGCGTCT	214,431,109	166
P06gInDel-39	TGGAGAGAGACAATGCCTGC	AGTGCAACTTTTCACCCCCA	214,482,771	206

Name	Upstream primer (5′-3′)	Downstream primer (5′-3′)	Position (bp)	Size (bp)
P06gInDel-40	GCCCCCAACTAACTCTAATTGC	ATTCCCACGTCTAGATTGCAT	214,511,256	193
P06gInDel-41	ATCAAACACTTGTATGGCATAAACC	TGAATTGCATGCTAAACCCATTGA	214,517,898	195
P06gInDel-42	TCCAAAGATGTCCAAGCCGT	GAAGGGAGGCTGAACACGTA	214,692,915	207
P06gInDel-43	GTTGTCTCTTATTTCACAAGTCTCA	CAACGGTGTGAAAACTAACGGA	214,878,098	267
P06gInDel-44	TCCCTATCACGTCCTCTGGTT	AGTTTGACTCCTTGTCGCACA	214,880,979	218
P06gInDel-45	TTTCTGGTGCAAGTTTTGACGA	CATGCGTGAATGAACTGAGAGT	214,882,397	151
P06gInDel-46	ATTGAATGTGGTTGCTTGCCT	TTGTTGTGTATGACGACTTACCT	214,885,975	255
P06gInDel-47	GATTTCTGAATTTCTGATCGCCA	ATTCTCCAAGTCAGGAAACAGTA	214,903,747	184
P06gInDel-48	AGATTTCTGAATTTCTGATCGCCA	AGTTTGGACAAGAAGAGTGACA	214,903,798	334
P06gInDel-49	ATTTGCTTGAGGCTTGAGACG	AGTTTTCTACCCGCTCACAGT	214,928,763	182
P06gInDel-50	CTCTTCTTCGGAGCCTCTGC	TGCTAGGTTGGATCATGTTGGA	214,929,424	224
P06gInDel-51	GCATTTTTACCCACGCTTTACTG	CACAATCAACAACAGCATGGC	214,930,476	117
P06gInDel-52	GCAGCCTTAGTGCCTACATGAAC	GCAGCCTTAGTGCCTACATGAAC	214,931,290	144
P06gInDel-53	AAAATCTTACATGTTCTACAAGCGA	ACGTGTTCCCAACAAAACGAC	214,941,431	284
P06gInDel-54	AGGTGTTTTAAGTGAATTTCGCCA	ACGAAGTTGATGCCCCATGA	214,942,822	199
P06gInDel-55	ACGACGAGCAAATCCAAAACAA	CCGAGAGATATTTTCAACGCCTT	214,946,579	250
P06gInDel-56	TTAATTTGATTGCTTGCCTAAGACT	AGTCAACTAACTAGTCAGGAGTGAA	214,950,959	246
P06gInDel-57	GCTCTTAGCTTGTAAGATAAAGGTT	AATTGTCTTTTGACCTAGTTTGACA	214,952,004	242
P06gInDel-58	TCCACACGCCGGGGTATTAT	TCAGATAGAGGGTACGGGGC	214,952,395	218
P06gInDel-59	TGACCACTAAAGTGGCCTGG	CTGATGCAGAAGCTTTAGCCG	214,995,429	181
P06gInDel-60	TTGCCTCTGAATAGCCGATTT	AGCCCTTTCTCTGAATAAACAATCA	215,010,750	245
P06gInDel-61	TGATTGTTTATTCAGAGAAAGGGCT	CGTAGGAAAAATGGTTCGTCTCC	215,011,008	165
P06gInDel-62	TCAGGTTCATCATCTAAACTCGT	TGGCTTAGTTTGGCATCTTTCTT	215,019,370	231
P06gInDel-63	GAGACAGTTGCAAGACCCGA	CACTTGTGAACCATTTCTTCGCT	215,020,538	179
P06gInDel-64	CTGACAGTCCTGATTCTGTGGA	AGCCTTCGTAAGTAGGCGAC	215,038,936	203
P06gInDel-65	TCATTTATTTCGAAACGGAGGGA	GAAGGTCATGCAGGTGTGAGT	215,049,179	148
P06gInDel-66	TGGAGCTAAAAGGAAAGGCACA	TTGGGTCACGACGTTAGCAT	215,097,259	216
P06gInDel-67	GCCCTTGGTCCAAAATCCCT	GTGAGTGTGGAGAACTGCGA	215,104,026	238
P06gInDel-68	GAAGAGTTCTGGCCTGAGGA	TGGTGCTCCCTCATTGGCTA	215,106,458	228
P06gInDel-69	AATTTGGCGAAAGGGCATGG	GCCCAAGTTCATCACCAACAC	215,111,984	185
P06gInDel-70	TCAATCAACTTTCGTGAGCCA	GCTCTCCACAACGTGGGTAA	215,115,346	172
P06gInDel-71	TGGCCAAGAACAAGATGGAC	GGTCACCAAAATAGTCTCGGGT	215,156,003	210
P06gInDel-72	CACGATTTTACTACCCCATA	GCGCATTCAAGAGTTCAT	215,170,558	151
P06gInDel-73	GAAAGCATCGAAACCACCCAG	TCCATCTGACCTCTCCATCGT	215,211,190	220
P06gInDel-74	TTCGTAAATATGCACACTCACATT	TCTATGGCCAAACGGCTACT	215,255,154	139
P06gInDel-75	TGTTCCTCTACTTTTCCACATTTCT	GGTTGTGTACCTGACCACCC	215,330,712	113
P06gInDel-76	TGTTTCTAGTTTTGCGATTA	CAAAATGACGAAAAGATGTA	215,335,602	199
P06gInDel-77	CTACTCCACAGCTTGAAACATCA	TCCGAGATCAAATGGGTAGACA	215,367,964	158
P06gInDel-78	AGGTCCAAGGTCCATTCTTGAC	CCACCACTCCACCTTCGTTT	215,419,568	111
P06gInDel-79	GAGGGTCTTCCTATGTTTGTCC	TGCACGAATGAACAGTAAACCA	215,419,831	250

Name	Upstream primer (5′-3′)	Downstream primer (5′-3′)	Position (bp)	Size (bp)
P06gInDel-80	TCATCATCCAAGGTATGTGGGG	TGAGTTTTCGGACATCGTGTGA	215,487,057	320
P06gInDel-81	TCATTCCCTTTTTCCAGCTCTG	ACAGGGGTGTTATGGGTGATT	215,498,386	208
P06gInDel-82	AGCTTCGCATGTTGAGAGAGT	GCTCCTACCTCCACTCACTTG	215,499,896	234
P06gInDel-83	GTGTAAGGCACTCACACCAGA	ACCTTTGACATGTCTACCGACT	215,561,790	121
P06gInDel-84	GCTTGTTTCCAACTTCATCCCC	ATCATTTTGTGCATGGTCAAGT	215,620,699	223
P06gInDel-85	CCAATGCACATAATCTAACGCA	GTCTTGTTCCCTATCCCAGCA	215,620,719	224
P06gInDel-86	TCCAATGCACATAATCTAACGCA	AGTTCATGCTAAGTCTTGTTCCCT	215,620,825	237
P06gInDel-87	GATGAATTGCGCATTGTTGCTG	AACACATGTAGACAAAACAGCTCA	215,628,077	184
P06gInDel-88	AGACTCCAGAACAGAAGTACCA	AGTTGGCTGTAGTGTTGGCA	215,630,850	250
P06gInDel-89	ATGCCAACACTACAGCCAACT	GCAAGTCTACTATTTCCCAGGTG	215,631,069	156
P06gInDel-90	GCACCTTCACCCCATTCCATA	GTCTTCTCTCTCCCCGTATTGG	215,633,948	205
P06gInDel-91	TATTGCACCCTACCGCTTGAG	GCTCCCTACCTCACATTATCCT	215,636,889	129
P06gInDel-92	GGTTGGCTATGATGGTGGTTTC	AGTAAGATTTGGAGGCGGCAT	215,669,032	126
P06gInDel-93	TCTCCATTGATACATTCAGGGCA	AGGAGATTTAGCGTGTGTCGT	215,669,309	122
P06gInDel-94	TGGGAAAGAATGTGATTGATGTGT	TAGGGCCCACTCAAAGATGC	215,669,384	176
P06gInDel-95	TCCTTCTTCGAACTCCACACG	ACGGAGTCTAAAGAAGAGTTCG	215,674,072	180
P06gInDel-96	CAAGGCAAGACTGCGAACAAT	GTGTGAAGTTTGAAAAAGGTTCGAT	215,674,480	199
P06gInDel-97	CAGCTAAAGCAGGCATGTCG	GGCTTCTTGGCAAAGCAGAAT	215,706,844	305
P06gInDel-98	AGCTGATTTTTCATCCTCATTTTCG	CGTTGCTTCTAACAGGTGCG	215,710,386	110
P06gInDel-99	GAGGGAGTTTCGTCATTCATGC	AGGATGTAAAGGGGATGTCTCG	215,741,617	234
P06gInDel-100	TGGATTGTGGTGTAGTGGTGA	TTGGAGCCCCGACTAATTTGG	215,769,174	143
P06gInDel-101	TTGTGGTGTAATGTGGGACTGT	AGGTGTCTGGTGTGGATATTGG	215,769,584	154
P06gInDel-102	ATCCGAAAACAATCCAATTCTCAA	GTGTATTTAGAGGCAGAGCTAGG	215,772,487	189

Table S3. Primer sequences used for candidate genes cloning.

Name	Upstream primer (5′-3′)	Downstream primer (5′-3′)	Length (bp)
gCapana06g002965	GGGTCCACACTATGTCATTTGG	TGTGGCAGCCCTAAACACAA	869
gCapana06g002967	ACTAGCGTTACTTGGGCACC	GTAAAGGTTGGCTACGCCCT	1351
gCapana06g002968-segment 1	TCACATGCCTCTTACTTTGCAC	TGGCGGACTTCAGTTACTTT	1423
gCapana06g002968-segment 2	AGCATATATAGCCGAAGTCCACA	TGTGTCGTTTAGTAGGATTGGT	1427
gCapana06g002968-segment 3	ATAGACAACGTTATTATAGAGA	TTAGCAGGACTGGATAAGAATA	1356
gCapana06g002968-segment 4	AGATCAAGGAGGACAAAA	AATAAAGGAGGTTACAAT	1333
gCapana06g002968-segment 5	AAAGAATGATAGAGGCAGTA	TTTAGGGTCGATTTCAGA	1527
gCapana06g002969	AGTTTTGTCATCATGGGATTTCCT	ACAGCCTTTCTACCTCATCAGA	1026

Table S4. Primer sequences used for qRT-PCR analysis.

Name	Upstream primer (5′-3′)	Downstream primer (5′-3′)	Length (bp)
qCapana06g002965	CCATGGTTGGTGAGACGAGA	TGTGCTCAAATGCGACCAAAA	157
qCapana06g002967	AGGGAGTCCAGTACGAGAGA	GCAAACCTGCTTGCGGATTA	156
qCapana06g002968	CCGATATGATGAGGCCCGTT	ATGTTTTGCAAGAAGGGCGG	144
qCapana06g002969	GACAGCCAGCTGAGTTTTGAT	CAGCATTGCTAGATCTTGATTCTGA	142
β-actin[52]	TGCAGGAATCCACGAGACTAC	TACCACCACTGAGCACAATGTT	95
